# Prehospital delay and thrombolysis rates in posterior circulation stroke: a multicenter Latin American study

**DOI:** 10.3389/fneur.2026.1941221

**Published:** 2026-10-15

**Authors:** Charles Huamani, Andrés A. Mercado-Pompa, Alfonso León-Mantero, Víctor Oré-Montalvo, Iván Baracaldo-Santamaría, Hebert Puente-Vila, Melissa Pérez, Rocío B. Pacheco-Elgueta, Max Molina-Jara, Andrés Diaz-Guzman, Hesed Virto-Farfán, Vanessa Cano-Nigenda, Antonio Arauz

**Affiliations:** 1 Universidad Andina del Cusco, Cusco, Peru; 2 Instituto Nacional de Neurología y Neurocirugía Manuel Velasco Suarez, Mexico City, Mexico; 3 Hospital Base de Valdivia, Valdivia, Chile; 4 Hospital Nacional Adolfo Guevara Velasco, Cusco, Peru; 5 Instituto Neurológico, Hospital Internacional De Colombia, Bogotá, Colombia; 6 Hospital Nacional Guillermo Almenara Irigoyen, Lima, Peru; 7 Hospital Nacional Carlos Seguín, Arequipa, Peru

**Keywords:** delay, Latin America, posterior circulation acute ischemic stroke, pre hospital, stroke

## Abstract

**Objective:**

We aimed to evaluate the delay to emergency arrival in adults with acute Posterior circulation ischemic strokes (PCS) and the likelihood of receiving intravenous thrombolysis compared to anterior circulation ischemic strokes (ACS) across six different hospitals in Latin America.

**Methods:**

This multicenter study in Latin America hospitals including three hospitals in Peru (Cusco, Arequipa, and Lima City), one in Colombia (Bogotá), one in Chile (Valdivia), and one in Mexico City. We analyzed adult patients treated between 2018 and 2023 with a confirmed diagnosis of ischemic stroke (IS). We evaluated demographic characteristics, initial NIHSS score, onset-to-door time (ODT), rate of intravenous thrombolysis (IVT), and other stroke-related characteristics. Medians with interquartile ranges (IQR) were calculated. IVT (yes/no) was evaluated using Firth penalized logistic regression including stroke circulation, age, sex, admission NIHSS, ODT, previous stroke, and previous evaluation at another hospital as covariates. Continuous predictors were modeled per year, NIHSS point, and ODT hour, respectively. Adjusted odds ratios (aOR) with 95% CI were reported, with *p* < 0.05 considered statistically significant.

**Results:**

A total of 1,460 patients were included, 501 (34.3%) of whom had PCS, with a median age of 70 years (IQR 59–80). We found significant differences in median ODT between PCS and ACS (13 h, IQR 5–46 vs. 8.5 h, IQR 4–20; *p* < 0.001) and in IVT rates. In the adjusted analysis, each additional hour of ODT was associated with lower odds of IVT (aOR 0.88, 95% CI 0.85–0.92), whereas stroke circulation was not independently associated with IVT (PCS vs. ACS: aOR 0.66, 95% CI 0.39–1.11). Higher admission NIHSS was associated with higher odds of IVT (aOR 1.06 per point, 95% CI 1.03–1.09).

**Conclusion:**

Patients with posterior circulation stroke had longer prehospital delays and lower observed rates of intravenous thrombolysis than those with anterior circulation stroke. However, stroke circulation was not independently associated with IVT after adjustment for ODT and other clinically relevant covariates. These findings highlight early recognition and reduction of prehospital delay as potential targets for improving access to reperfusion therapy in Latin America.

## Introduction

Ischemic stroke (IS) is associated with high morbidity and mortality, making it a significant public health problem in Latin America (1–3). In 2021, an estimated 11.9 million incident strokes and 7.3 million stroke-related deaths occurred globally, with IS accounting for 65.3% of incident strokes. In Latin America and the Caribbean, approximately 554,000 incident strokes and 279,000 stroke-related deaths occurred in the same year (2–4). Posterior circulation stroke (PCS) represents approximately 20–25% of IS, with the remaining majority involving the anterior circulation (ACS) (5). Although stroke burden is substantial across Latin America, regional population-based data specifically distinguishing the burden of PCS and ACS remain limited. The therapeutic window for reperfusion therapies is very narrow, and several limitations still exist across the region (6). One of the most consistent limitations in all countries is the delay in arriving at the emergency services for patients with IS (7). Longer prehospital delays reduce access to time-dependent reperfusion therapies and are associated with worse functional outcomes. In a prospective Mexican cohort of patients with IS, only 20.9% arrived within 3 h of symptom onset, and the time from symptom recognition to the decision to seek medical attention accounted for more than half of the onset-to-door time. PCS may be particularly affected because prehospital delays are generally longer than in ACS (8). These delays have multiple causes, including distance from the hospital, limited accessibility, cultural beliefs that the patient might improve on their own, and, especially, failure to recognize the symptoms (9, 10). Posterior circulation ischemic strokes (PCS) present different, less specific signs and symptoms, which can contribute to diagnostic delays and reduce opportunities to provide reperfusion therapies (11).

The benefit of thrombolysis in PCS is well established (12), however, delayed hospital arrival may reduce the opportunity to receive reperfusion therapy (13, 14). However, differences in prehospital delay between PCS and ACS have not been specifically evaluated in Latin America, where geographic, socioeconomic, and healthcare access barriers may influence timely hospital arrival. Identifying whether PCS is independently associated with delayed arrival and lower access to IVT could help define targets for public awareness, prehospital recognition, and stroke-care pathways in the region. Therefore, our objective was to evaluate the delay to emergency arrival in adults with acute PCS and the likelihood of receiving intravenous thrombolysis compared with ACS across six different hospitals in Latin America.

## Methods

### Study design and population

An observational, cross-sectional, analytical, retrospective, and multicenter study was conducted in 3 hospitals in Peru (Cusco, Arequipa, Lima), one in Colombia (Bogotá), one in Chile (Valdivia), and one in Mexico City (Figure 1). We included adult patients admitted between January 2018 and December 2023. At each participating center, potentially eligible patients were retrospectively identified from institutional records using ICD-10 codes compatible with cerebral infarction (e.g., I63.9, I69.3, and related codes). Medical records of the identified patients were subsequently reviewed at each center, and only patients meeting the predefined eligibility criteria for acute ischemic stroke were included. The number of records initially screened before application of the eligibility criteria was not collected. Hospitals were selected if they were referral hospitals with 24/7 care capacity and availability for intravenous thrombolysis (IVT). The hospitals were chosen for convenience; three of the six were located outside the country’s capitals. All centers have neurologists who perform essential stroke management and have local guidelines prioritizing IVT. The protocol was approved by the ethics committee of each site.

**Figure 1:**
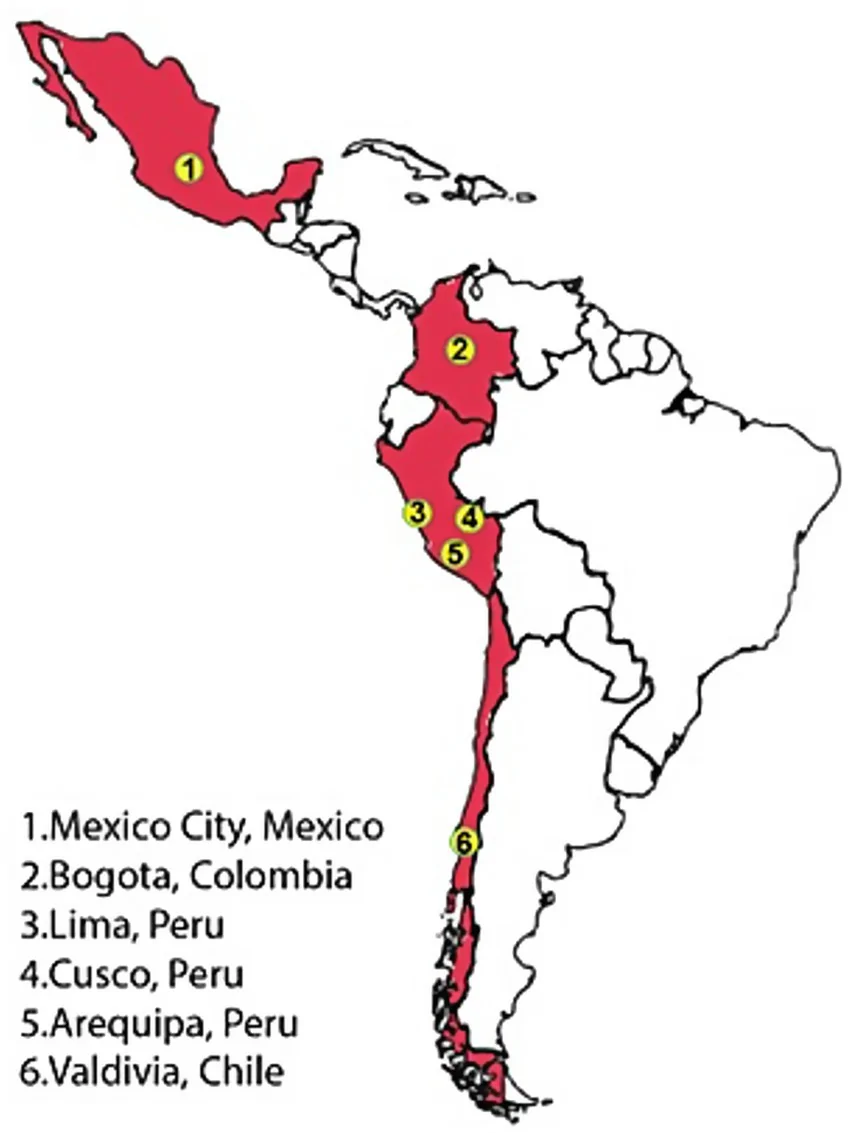
Location of participating hospitals.

Patients who met the following criteria were selected: adult patients admitted to emergency services with suspected stroke, diagnosed by a neurologist, and confirmed by imaging studies (CT or MRI). Those with incomplete clinical information, either on stroke data or who had not been evaluated using the National Institutes of Health Stroke Scale (NIHSS), were excluded. The number of patients excluded for each individual exclusion criterion was not recorded during the retrospective data collection.

Although there are criteria for IVT in patients who arrive in extended windows (up to 9 h from symptom onset) (6), patients treated with IVT under extended-window criteria were excluded from the study, regardless of the selection method used. A structured data collection form and dictionary for correct variable entries were designed and shared with each hospital. Some sites have specific electronic systems for registering stroke patients (e.g., Redcap), and some have certification as basic or advanced stroke care centers by the World Stroke Organization (WSO).

The funder did not influence the results/outcomes of the study despite author affiliations with the funder.

### Data and outcome

Demographic characteristics (gender, age), vascular risk factors, onset to door time (ODT), affected circulation: posterior circulation stroke (PCS) or anterior circulation stroke (ACS), NIHSS score on admission, and received treatment (IVT or conservative treatment), were evaluated. PCS was defined as an infarction in the territory of the vertebrobasilar system (vertebral, basilar, and posterior cerebral arteries and their branches), which could affect the brainstem, cerebellum, thalamus, or occipitoparietal lobes (15).

For PCS, due to the low sensitivity of cranial tomography studies, the records were based on the neurologist’s diagnosis. When the infarction was not evident on CT, the approach was supplemented with MRI. No image review was performed, and the diagnosis of PCS was based on the protocols of each center.

The “therapeutic window” was defined as arrival at the hospital within 4.5 h from symptom onset, as this is the usual time for intravenous thrombolysis. For wake-up strokes, estimated symptom onset was defined as the midpoint between the last-known-well time and the time of symptom recognition. This estimated onset time was used to calculate ODT and to classify patients according to whether they arrived within or beyond 4.5 h of estimated symptom onset.

The other variables included were whether the patient attended another care center before reaching the primary hospital, whether the patient had a wake-up stroke, length of hospital stay, and death at 90 days.

### Statistical analysis

Descriptive analyses were performed for the collected variables (risk factors, NIHSS on admission, ODT, country and site of origin, etc.). Qualitative variables were presented as absolute and relative values (n and %), and quantitative variables were presented as medians with interquartile ranges (IQR) due to their non-normal distribution, assessed using the Shapiro–Wilk test.

The two primary outcomes were: ODT by affected circulation (PCS vs. ACS) and IVT vs. conservative treatment.

For the analysis of prehospital delay, ODT was summarized using medians and interquartile ranges because of its non-normal distribution. Differences in ODT between PCS and ACS were initially assessed using the Mann–Whitney *U* test. ODT was also dichotomized as <4.5 versus ≥4.5 h to evaluate factors associated with arrival within the conventional therapeutic window using logistic regression. In addition, median regression was performed with ODT as a continuous outcome to directly quantify the adjusted difference in prehospital delay between PCS and ACS. The median regression model included stroke circulation, wake-up stroke, previous evaluation at another hospital, country, admission NIHSS, age, and sex.

For IVT, crude odds ratios (cOR) were estimated using conventional logistic regression. Covariates for the adjusted model were selected based on their clinical relevance and plausible association with IVT and were retained irrespective of statistical significance in the crude analysis. Because the conventional multivariable logistic regression model showed evidence of separation when ODT was included as a continuous predictor, adjusted estimates were obtained using Firth penalized logistic regression. The IVT model included stroke circulation (PCS vs. ACS), age (continuous, per year), sex (male vs. female), admission NIHSS (continuous, per point), ODT (continuous, per hour), previous stroke (yes vs. no), and previous evaluation at another hospital (yes vs. no). All covariates were entered simultaneously into the adjusted model and retained irrespective of their statistical significance. As a sensitivity analysis, a separate Firth penalized logistic regression model was performed among patients arriving within 4.5 h of symptom onset, using the same covariates as the primary IVT model. A separate adjusted model was not performed among patients arriving ≥4.5 h because only 15 patients in this subgroup received IVT, which was considered insufficient for reliable multivariable estimation.

Statistical significance was defined as *p* < 0.05. All analyses were conducted in STATA v.16.1 statistical software. This study followed the STROBE guideline recommendations.

### Ethics statement

The main protocol was approved by the Instituto Nacional de Neurología y Neurocirugía in Mexico (ID 47/23). In addition, approval was obtained from the ethics and/or research committee at each participating site.

## Results

A total of 1,460 patients were included, of whom 796 (54.5%) were men, with a median age of 70 years (IQR 59–80). Patients with PCS were older than those with ACS (74 years [IQR 63–82] vs. 70.5 years [IQR 60–78]; *p* < 0.05), whereas the proportion of male patients was similar between groups (57.3% vs. 53.1%; *p* = 0.125). PCS patients had a lower NIHSS score on admission (PCS: 5 (IQR 3–9); ACS: 9 (IQR 5–16); *p* < 0.001). The baseline characteristics are listed in Table 1.

**Table 1:** Baseline characteristics of the population of 6 hospitals in 4 different countries.

Characteristics	Total (*n* = 1,460)	ACS (*n* = 959)	PCS (*n* = 501)	*p*-value
Age (years)	70	70.5	74	0.001
	(IQR 59–80)	(IQR 60–78)	(IQR 63–82)	
Male	796	509	287	0.125
	(54.5%)	(53.1%)	(57.29%)	
Hypertension	59.0%	64.2%	50%	<0.001
Diabetes	21.5%	20.7%	22.8%	0.513
Atrial fibrillation	18.2%	21.2%	12.9%	0.007
Smokers	9.5%	10.4%	7.9%	0.280
Previous stroke	22.2%	23.4%	20.1%	0.306
Country				
Peru	718 (49.2%)	63.4%	36.2%	<0.001
Mexico	448 (30.7%)	64.9%	35.0%	
Colombia	145 (9.9%)	82.7%	17.2%	
Chile	149 (10.2%)	60.4%	39.6%	
Arrived to another hospital before	25.7%	23.9%	28.7%	0.162
Admission NIHSS	7 [4–14]	9 [5–16]	5 [3–9]	<0.001
Wake-up stroke	13.9%	15.7%	9.6%	0.006

NIHSS, National Institutes of Health Stroke Scale; PCS, posterior circulation strokes; ACS, anterior circulation strokes; IQR, interquartile range.

### Stroke delays

Patients with PCS experienced longer prehospital delays (PCS: 13 h (IQR 5–46); ACS: 8.5 h (IQR 4–20); *p* < 0.001) (Figure 2), even considering only those arriving within the first 24 h (Table 2). However, among those who arrived within the therapeutic window (<4.5 h), they had the same median ODT (*p* = 0.773), although the proportion of PCS patients within the therapeutic window was lower (PCS: 84, [17.1%] vs. ACS: 290 [30.9%]; *p* < 0.001, Table 2). The ODTs by circulation (PCS vs. ACS) were evaluated across the variables described in Table 1, with generally longer ODT among PCS patients across most strata (Supplementary Table e1). In median regression, PCS was associated with a 4.11-h longer median ODT compared with ACS after adjustment for wake-up stroke, previous evaluation at another hospital, country, admission NIHSS, age, and sex (95% CI 1.78–6.44; *p* = 0.001) (Supplementary Table e2).

**Figure 2:**
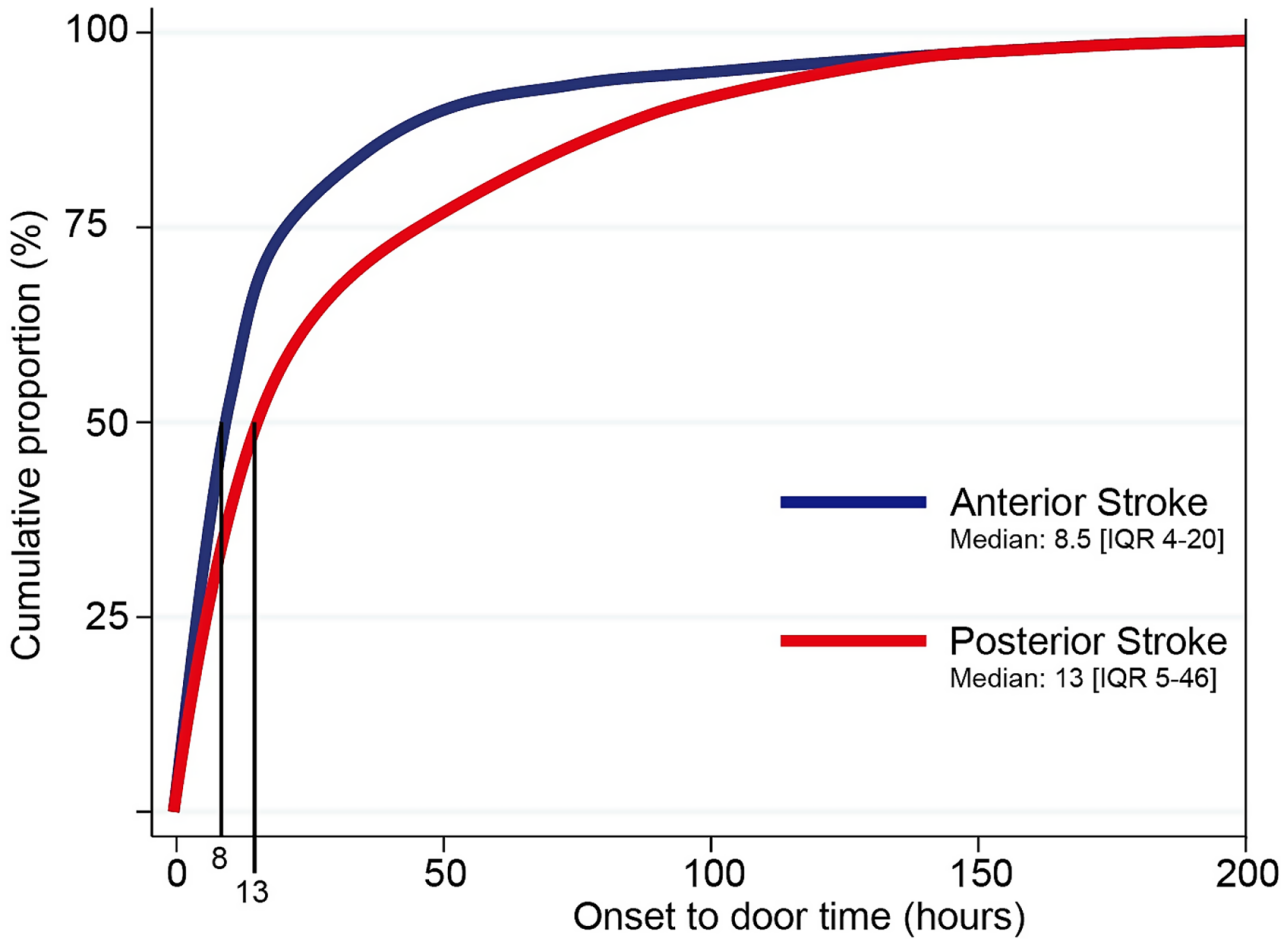
Association of prehospital delay and stroke localization (ACS versus PCS).

**Table 2:** Prehospital delay according to stroke circulation.

Onset-door	Total	ACS	PCS	*p*-value
All patient				
*N*	1,429 (100%)	938 (100%)	491 (100%)	
Time (hours)*	10 [4–24]	8.5 [4–20]	13 [5–46]	<0.001
Within <24 h				
*N*	1,064 (74.5%)	750 (79.9%)	314 (63.9%)	
Time (hours)*	7 [3–13]	6 [3–12]	9 [4–15]	<0.001
Within <4.5 h				
*N*	374 (26.2%)	290 (30.9%)	84 (17.1%)	
Time (hours)*	2.3 [1.5–3.4]	2.3 [1.2–3.5]	2.5 [1.5–3.0]	0.773

Median with IQR, interquartile range; PCS, posterior circulation strokes; ACS, anterior circulation strokes.

Patients with lower odds of arriving within 4.5 h were those with PCS (aOR 0.57, 95% CI 0.40–0.80; *p* = 0.001), wake-up stroke (aOR 0.15, 95% CI 0.08–0.28; *p* < 0.001), and previous evaluation at another hospital (aOR 0.24, 95% CI 0.17–0.34; *p* < 0.001). Higher admission NIHSS was associated with greater odds of arrival within 4.5 h (aOR 1.04 per point, 95% CI 1.02–1.07; *p* < 0.001). Other variables related to the time window are presented in Table 3.

**Table 3:** Probability of arrival within the therapeutic window.

Onset-door time <4.5 h	cOR (CI95%)	aOR (CI95%)^*^	*p*-value
PCS	0.46 (0.35–0.61)	0.57 (0.40–0.80)	0.001
Wake-up stroke	0.20 (0.11–0.37)	0.15 (0.08–0.28)	<0.001
Arrived to another hospital before	0.32 (0.23–0.44)	0.24 (0.17–0.34)	<0.001
Country			
Peru	Ref.	Ref.	0.609
Mexico	0.79 (0.60–1.05)	0.91 (0.55–1.52)	0.014
Colombia	1.39 (0.94–2.04)	1.66 (1.05–2.64)	0.039
Chile	0.97 (0.65–1.44)	1.65 (1.03–2.62)	
NIHSS on admission	1.05 (1.03–1.06)	1.04 (1.02–1.07)	<0.001
Age	1.01 (1.01–1.02)	1.00 (0.99–1.01)	0.752
Male	1.41 (1.11–1.79)	1.38 (0.99–1.85)	0.052
Hypertension	1.33 (1.04–1.70)		
Diabetes	0.63 (0.47–0.85)		
Atrial fibrillation	1.58 (1.16–2.17)		
Previous stroke	0.98 (0.71–1.35)		

PCS, posterior circulation strokes.

^*^Covariates included in the adjusted model were selected based on their clinical relevance and plausible association with timely hospital arrival and were retained irrespective of statistical significance.

In sensitivity analyses excluding Peru or Mexico, PCS remained independently associated with lower odds of arrival within 4.5 h (excluding Peru: aOR 0.36, 95% CI 0.17–0.73; *p* = 0.005; excluding Mexico: aOR 0.51, 95% CI 0.36–0.72; *p* < 0.001) (Supplementary Table e3).

### Rate and probability to IV thrombolysis

Among patients arriving within 24 h, PCS patients received IVT less frequently than ACS patients (9.6% vs. 18.3%; *p* < 0.001). Among patients arriving within 4.5 h, IVT rates were 32.1 and 43.8%, respectively (*p* = 0.056) (Table 4).

**Table 4:** Thrombolytic therapy, length of hospital stay, and 90-day mortality among PCS and ACS patients.

Outcome	Total	ACS	PCS	*p*-value
Thrombolytic therapy				
Patients arrived <24 h	167 (15.7%)	137 (18.3%)	30 (9.6%)	<0.001
Patients arrived <4.5 h	154 (41.2%)	127 (43.8%)	27 (32.1%)	0.056
Days of hospital stay^*^	8 [4–14]	8 [3–14]	9 [5–14]	0.075
Death at 90 days	9.4%	11.6%	5.9%	0.001

PCS, posterior circulation strokes; ACS, anterior circulation strokes.

^*^Median with interquartile range.

In the adjusted Firth penalized logistic regression model, longer ODT was associated with lower odds of IVT (aOR 0.88 per additional hour, 95% CI 0.85–0.92; *p* < 0.001). PCS was not independently associated with IVT (aOR 0.66, 95% CI 0.39–1.11; *p* = 0.122). Higher admission NIHSS was associated with higher odds of IVT (aOR 1.06 per point, 95% CI 1.03–1.09; *p* < 0.001), whereas previous stroke (aOR 0.46, 95% CI 0.26–0.82; *p* = 0.008) and previous evaluation at another hospital (aOR 0.49, 95% CI 0.30–0.80; *p* = 0.005) were associated with lower odds of IVT. The other variables associated with IVT treatment are presented in Table 5. In the sensitivity analysis restricted to patients arriving within 4.5 h, PCS was not independently associated with IVT (aOR 0.88, 95% CI 0.45–1.68; *p* = 0.691). Higher admission NIHSS was associated with higher odds of IVT (aOR 1.10 per point, 95% CI 1.06–1.15; *p* < 0.001), whereas previous stroke was associated with lower odds of IVT (aOR 0.37, 95% CI 0.17–0.74; *p* = 0.006) (Supplementary Table e4).

**Table 5:** Probability of thrombolysis.

Variable	Crude OR (CI 95%)	Adjusted OR^*^ (CI 95%)	*p*-value
Onset door time (ODT)	**0.65 (0.59–0.71)**	0.88 (0.85–0.92)	<0.001
PCS	0.41 **(0.24–0.72)**	0.66 (0.39–1.11)	0.122
NIHSS on admission	1.06 **(1.03–1.10)**	1.06 **(1.03–1.09)**	<0.001
Previous stroke	0.29 **(0.13–0.64)**	0.46 **(0.26–0.82)**	0.008
Arrived to another hospital before	0.28 **(0.13–0.59)**	0.49 (0.30–0.80)	0.005
Male	1.32 (0.83–2.08)	1.02 (0.69–1.53)	0.854
Age (years)	1.01 (0.99–1.02)	0.99 (0.97–1.01)	0.085
Hypertension	1.05 (0.661.69)	–	
Atrial Fibrillation	1.50 (0.87–2.59)	–	
Diabetes	0.30 **(0.14–0.67)**	–	
Smoke	0.58 (0.22–1.49)	–	
Country			
Peru	Ref.	–	
Mexico	1.1 (0.7–1.6)		
Colombia	0.9 (0.6–1.7)		
Chile	0.5 (0.2–0.9)		

^*^Adjusted estimates were obtained using Firth penalized logistic regression because the conventional multivariable logistic model showed evidence of separation when ODT was included as a continuous predictor. Bold values indicate statistically significant associations.

## Discussion

The main strength of our study is that it is a multicenter study involving several Latin American countries, which provides valuable information on the acute management of ischemic stroke (IS) in our region. Although our primary objective was to evaluate posterior circulation strokes, our findings showed that the time metrics for treatment (onset-to-door times) in both anterior and posterior circulation strokes remain suboptimal for the acute management of IS. This suggests that there is a neglected public health problem that requires a multidisciplinary approach that can identify the key issue in the management of patients with acute ischemic stroke, especially those with posterior circulation involvement. Therefore, strategies are urgently needed for the early recognition of IS in the general population and for the identification of hospitals with adequate infrastructure for IS management.

In our study, these delays corresponded to a 4.5-h difference in the time from symptom onset to hospital arrival for patients with PCS and ACS. As in many other countries, stroke is highly prevalent in Latin America, so there are several actions aimed at improving care (2), and although part of the study was developed in the context of the COVID-19 pandemic, which increased pre-hospital delay by an average of 1 h and generated worse hospital outcomes (16, 17), our results indicate that the delay associated with the location of the affected circulation is several times greater. This difference is enormous compared with other reports, in which the average difference is approximately 27 min (a systematic review including 5,395 patients from 15 studies) (18). Although prehospital delays vary across populations, they place us among the worst cases in terms of timely attention to a severe problem. Importantly, PCS was also not independently associated with IVT in the sensitivity analysis restricted to patients arriving within 4.5 h, although the confidence interval was wide and this finding should be interpreted cautiously. Longer ODT was independently associated with lower odds of receiving IVT. After adjustment for ODT and other clinically relevant covariates, stroke circulation was not independently associated with IVT. These findings suggest that stroke circulation may not independently determine IVT treatment, while timely hospital arrival appears to play a more important role (15). The effectiveness of reperfusion interventions, such as IVT or mechanical thrombectomy, reported in Latin American patients are similar to those described in higher-income countries (19, 20), and eventually in-hospital times could improve and achieve care goals (21); our study shows that the proportion of patients who manage to access IVT is similar to that described in other reports. For example, approximately 30% of patients arriving within ≤4.5 h, approximately 30% meet criteria for IVT (22). A study in China showed that the proportion of IVT among all patients attending a center may vary depending on whether it is a secondary (6.4%) or tertiary care center (5.4%) (23), results that are consistent with our study. The observed difference in thrombolysis rates was attenuated after adjustment for prehospital delay and other clinically relevant covariates. The proportion of thrombolyzed patients is in accordance with that reported in other regions suggests that the main critical node to achieve thrombolysis is in the early identification of patients, while the centers comply by providing care in relation to the protocolized times. Our findings suggest that posterior circulation involvement should not be considered a barrier to reperfusion therapy itself. Instead, the apparent treatment disparity is largely explained by delayed hospital arrival, highlighting prehospital recognition as the principal target for intervention.

It has already been described that the Spanish-speaking population has less knowledge about stroke and tends to underestimate its severity. (24–26) One of the factors that could contribute to this is that several names for stroke are used, such as “*accidente cerebrovascular*,” “*ataque cerebral*,” “*enfermedad cerebrovascular*,” “*ictus*,” or colloquially named as “*derrame cerebral*” (27). Another factor is that most strategies for prehospital diagnosis are in English or partially in English (FAST, RACE, CAMALEON) (28). Although these differences have been studied and maybe have implications for care and treatment (29), they are not the only factors since racial, ethnic, social, cultural and even discriminatory aspects may be involved (30, 31), aspects that should be further studied. Finally, improving prehospital attention also has to do with changes in the organization of health services and the certification of stroke centers (32, 33), because these centers would be responsible for training the population in their environment, which is still a challenge pending attention in Latin America.

This study has some limitations. Silent ischemic lesions may not be identified using the imaging protocols currently available at some participating centers, although MRI was performed when clinically indicated. Patients treated with IVT under extended-window criteria were excluded from the analysis because extended-window treatment protocols were not uniformly available across participating centers. Consequently, our findings should not be extrapolated to patients treated under extended-window selection protocols. The total number of records initially screened, and the number excluded according to each individual exclusion criterion were not collected and could not be reliably reconstructed. Thus, the proportion of potentially eligible patients included and the potential selection bias related to incomplete data cannot be determined, which should be considered when interpreting the sample size and generalizability of our findings. The participating hospitals were selected by convenience and were referral centers with 24/7 stroke care, which may introduce selection and referral bias and limit the generalizability of our findings to less specialized or rural settings. In addition, standardized data on door-to-needle time, prehospital stroke-code activation, specific arterial territory or occlusion subtype in PCS, and contraindications to IVT were not systematically available across centers. No centralized imaging adjudication was performed. These limitations restrict our ability to determine the mechanisms underlying differences in IVT administration. Finally, not all Latin American countries were not included. Nevertheless, this study is one of the largest designed in our region, where the problems may differ slightly but share many common difficulties; thus, the conclusions approximate the problems seen in Latin America.

In conclusion, patients with PCS experienced longer prehospital delays and had lower observed rates of IVT than patients with ACS. Longer ODT was independently associated with lower odds of receiving IVT, whereas stroke circulation was not independently associated with IVT after adjustment. These findings highlight the importance of improving early stroke recognition and reducing prehospital delays to facilitate timely access to reperfusion therapy in Latin America.

## Data Availability

The raw data supporting the conclusions of this article will be made available by the authors upon reasonable request and subject to appropriate authorization.
