# Supplementary material for: Prehospital delay and thrombolysis rates in posterior circulation stroke: a multicenter Latin American study
Source: Front Neurol. 2026 Oct 15;17:1941221. doi: 10.3389/fneur.2026.1941221 (PMC13716858; doi:10.3389/fneur.2026.1941221)
Supplement: Supplementary file 1 [file Table_1.docx]

**Tabla e1. Difference in arrival time to hospital (Onset-door time in hours) among different affected circulation (PCS: posterior circulation strokes vs ACS: anterior circulation strokes)**

| **Onset-door time** | **Total** | **p value** | **ACS** | **PCS** | **p value** |
| --- | --- | --- | --- | --- | --- |
| **Age ****  **≤70 years**  **≥ 70** | 12 [5-29]  9 [4-24] | <0.001 | 9 [4-24]  8 [4-22] | 23 [7-64]  12 [5-26] | <0.001  <0.001 |
| **Gender**  **Femal: Female, male**  **Male** | 9 [4-24]  12 [5-26] | <0.001 | 7 [3-19]  10 [4-24] | 12 [5-48]  20 [8-48] | <0.001  <0.001 |
| **Hypertension**  **No**  **Yes** | 12 [5-27]  10 [4-24] | 0.034 | 9 [4-24]  8 [4-22] | 17 [7-48]  16 [6-48] | <0.001  <0.001 |
| **Diabetes**  **No**  **Yes** | 10 [4-24]  14 [6-27] | <0.001 | 8 [3-21]  11 [5-24] | 14 [6-48]  21 [9-48] | <0.001  <0.001 |
| **Atrial Fibrillation**  **No**  **yes** | 12 [5-26]  8 [3-18] | <0.001 | 9 [4-24]  7 [2-18] | 18 [7-48]  10 [5-23] | <0.001  0.126 |
| **Previous stroke**  **No**  **Yes** | 12 [4-26]  10 [5-23] | 0.126 | 8 [4-24]  8 [4-20] | 19 [7-48]  12 [6-27] | <0.001  0.054 |
| **Country**  Peru  Mexico  Colombia  Chile | 10 [4-24]  12 [5-34]  9 [3-24]  12 [4-32] | <0.001* | 8 [4-20]  9 [4-24]  7 [3-24]  8 [4-24] | 13 [5-46]  24 [10-72]  24 [11-26]  20 [6-48] | <0.001  <0.001  0.001  0.006 |
| **Arrived at another healthcare facility** |  |  |  |  |  |
| **No**  **Yes** | 7 [3-18]  17 [7-44] | <0.001 | 6 [3-18]  13 [6-25] | 9 [4-24]  24 [12-72] | <0.001  <0.001 |
| **Wake-up stroke**  **No**  **yes** | 8 [4-24]  13 [8-20] | <0.001 | 7 [3-24]  13 [8-18] | 14 [5-47]  14 [10-28] | <0.001  0.166 |

* The Kruskal–Wallis test was performed, for the other variables, the Mann–Whitney U test was used.

** We choose the median of the population as a cutoff point

**Table e2.** Median regression analysis of factors associated with onset-to-door time

| Variable | Adjusted difference in median ODT, hours (95% CI) | p value |
| --- | --- | --- |
| Posterior circulation stroke | **4.11 (1.78 to 6.44)** | **0.001** |
| Wake-up stroke | 5.17 (2.35 to 7.99) | <0.001 |
| Previous evaluation at another hospital | 8.08 (5.86 to 10.30) | <0.001 |
| Country |  |  |
| Peru | Reference |  |
| Mexico* | 0.55 (−2.76 to 3.86) | 0.744 |
| Colombia* | −1.30 (−4.46 to 1.87) | 0.421 |
| Chile* | −0.19 (−3.24 to 2.86) | 0.901 |
| Admission NIHSS, per point | −0.19 (−0.33 to −0.04) | 0.014 |
| Age, per year | −0.02 (−0.09 to 0.05) | 0.605 |
| Male sex | −2.20 (−4.22 to −0.19) | 0.032 |

ODT: onset-to-door time; NIHSS: National Institutes of Health Stroke Scale. Coefficients represent the adjusted difference in median ODT, expressed in hours. Positive coefficients indicate longer median ODT and negative coefficients indicate shorter median ODT. The model included 1,045 patients.

**Table e3.** Probability to arrive on “therapeutic window” without Peru or Mexico

|  | **Without Peru** | | **Without Mexico** | |
| --- | --- | --- | --- | --- |
| **Onset-door time <4.5h** | aOR (CI95%)* | P value | aOR (CI95%)* | P value |
| **PCS** | 0.36 (0.17-0.73) | 0.005 | 0.51 (0.36-0.72) | <0.001 |
| **Wake-up stroke** | 0.23 (0.10-0.52) | <0.001 | 0.17 (0.09-0.31) | <0.001 |
| **Arrived to another hospital before** | 0.29 (0.18-0.46) | <0.001 | 0.32 (0.22-0.46) | <0.001 |
| **NIHSS on admission** | 1.04 (1.01-1.08) | 0.022 | 1.04 (1.02-1.06) | <0.001 |
| **Age** | 1.04 (1.01-1.08) | 0.64 | 0.99 (0.98-1.01) | 0.865 |
| **Male** | 1.40 (0.87-2.23) | 0.161 | 1.50 (1.10-2.03) | 0.009 |

PCS: posterior circulation strokes

* Covariates included in the adjusted model were selected based on their clinical relevance and plausible association with timely hospital arrival and were retained irrespective of statistical significance

**Table e4.** Factors associated with intravenous thrombolysis among patients arriving within 4.5 hours of symptom onset.

|  | Adjusted OR *  (IC 95%) | p value |
| --- | --- | --- |
| Onset Door Time (ODT) | 0.84 (0.68-1.04) | 0.116 |
| PCS | 0.88  (0.45-1.68) | 0.691 |
| NIHSS on admission | 1.10  **(1.06-1.15)** | <0.001 |
| Previous stroke | 0.37  **(0.17-0.74)** | 0.006 |
| Arrived to another hospital before | 0.61  (0.32-1.19) | 0.117 |
| Male | 0.59  (0.35-1.00) | 0.052 |
| Age (years) | 0.99  (0.97-1.01) | 0.117 |

* Adjusted estimates were obtained using Firth penalized logistic regression, consistent with the primary IVT model.
